# The Management of Crèches and Day Nurseries

**Published:** 1912-04-20

**Authors:** 


### THE MANAGEMENT OF CRÈCHES AND DAY NURSERIES.

### A Critical Comparison, with Suggested Reforms.

The existence in London of a large number of crèches and day nurseries, where infants and children can be looked after during the day, is a fact that has not, perhaps, attracted hitherto the attention which it deserves. The number of babies received daily must be very considerable.

There are between fifty and sixty crèches in London, providing food and care during the day for the children of women who go out to work. Some are run in connection with missions or churches, others are supervised by committees, others are managed privately. The effectiveness of their work varies also : some are excellently managed, others are lacking in many essential points, while a few can only be described as a positive danger to those unfortunate children whose mothers use them.

Some of the facts in connection with the management of day nurseries were recently brought to the notice of the Charity Organisation Society, and it was decided to make an inquiry, through the forty district committees of the Society in London; to tabulate the replies received; to hold a meeting to which persons interested might be invited to discuss the matter; and, finally, to take steps, if possible, to assist in remedying existing defects, if such were found. It is with this inquiry and with the results of it that we wish to deal.

Visits were paid to crèches and day nurseries by experienced visitors from the district committees of the Society. Thirty-eight crèches and day nurseries were fully reported on, and for purposes of comparison were divided into four classes :—

| | | | | |
|---|---|---|---|---|
| Class I. | Model | 3 crèches | | |
| ,, II. | Good | 14 | ,, | 38 |
| ,, III. | Fair only | 14 | ,, | |
| ,, IV. | Unsatisfactory | 7 | ,, | |

The basis of selection was the whole series of points, social and medical, dealt with in the inquiry. It is found that in a model crèche precautions against the use of it by those who have no claim upon it are adequate, the medical supervision is sufficient, the whole arrangements for feeding, washing, and dressing the children are good, the accommodation is ample, and the education of the mother is not forgotten. Class II. crèches are those which are well managed, but not perfect in one or other of these respects. In Class III. are to be found those crèches which could not fairly be described as completely unsatisfactory, but which are lacking in many essential matters. Class IV. crèches are those in serious need of drastic alteration, some, indeed, being so badly managed as actually to prove dangerous to the children received in them. Such a division must be somewhat arbitrary, but the numbers are perhaps not without value.

The National Society of Day Nurseries has fifteen affiliated crèches in the Charity Organisation Society's area. Of these four were not visited by district committees, and are consequently not included in the above figures. All three crèches in Class I. are affiliated to this Society.

In many day nurseries little effort is made to ascertain that mothers sending children really have to go out to work. In some cases, however, recommendations are required, and in others home visits are paid. In a few cases efforts are made to give some instruction to the mothers as to the care of their children, but in this respect not very much is accomplished. Medical inspection often means little more than that a doctor can be called in if required. One untrained matron was found who doses children whenever she thinks fit. It need hardly be added that the absence of effective medical control has resulted in many outbreaks of illness. In the matter of clothing there is much divergence of practice; a complete change is provided in some cases, in others pinafores are put over the children's own clothes. In one crèche, for some inscrutable reason, the babies are provided with knitted caps, which are used indiscriminately, with results that may easily be imagined.

In the matter of the washing of babies there is also diversity of opinion : in one day-nursery the children are not bathed, because, according to the matron, the mothers object. It may be added that this is one of the crèches about which there have been many complaints in its neighbourhood. In many crèches the objectionable long-tube bottle, recently forbidden by law in France, is in use, and "dummies," so vigorously denounced by Mr. John Burns, are very commonly found. The food itself varies from milk and water to the patent food made up by a local chemist. The "pound" mattress—a convenient way of disposing of sleeping children, and an admirable method of encouraging infection—is used in many crèches; some, too, are close and dirty, and occasionally actually insanitary.

Crèches at present are not subject to inspection by any authority, though the medical officer of health visits some of them. In the crèches affiliated to the National Society of Day Nurseries an annual report from the medical officer of health is required, or, in default of it, reports from the visiting physician and from the sanitary authority. The London County Council received a report on crèches and day nurseries in 1904, and this report was brought up to date in 1908. Details of management are given, and an extension of the system is recommended, but no existing defects are mentioned; surely these should be eradicated before the system is extended.

Some quotations from the reports on a few crèches may be given :—

No. 1 (West London).—"Five children in bed in a small back bedroom, the air very bad, windows and door tight shut."

"No questions are asked the mothers so long as they can pay their money down (6d. a day). There is no regular doctor to visit : if a child looks ill the matron refuses it. The children are not washed."

No. 2 (South London).—Extracts from accounts given by mothers :—

"Bottles were taken from babies' mouths, and passed on to others. The milk was very thin, much mixed with water, and the sugar in a sediment at the bottom."

"Mrs. B. has two children at the crèche. No inquiries are made. She and her husband both work, and earn 39s. a week."

The local committee also report that a doctor asked them to call on a woman whose child had been two weeks at the crèche. It was sent home very ill, and died, the doctor certifying that dirty milk was the cause.

It is a relief to turn from such quotations as these to the account of the Fulham Day Nursery. Here are some extracts from it :—

"The matron is a trained nurse. Crèche clothes are worn, and the children's own clothes are fumigated.

Separate clothes are kept for each child, and separate towels and combs used."

"The babies are bathed daily. Separate bottles and teats, numbered and washed in separate basins, and sterilised daily, are kept for each baby."

The general opinion of the district committees of the Society on the subject of the desirability of establishing and maintaining crèches is of interest. They regard crèches without very much enthusiasm, because they feel that, at their best, they are a poor substitute for a mother's care and attention. The ideal is that every woman should be able to look after her own baby at home. Unfortunately, this is not always possible, and, in that case, a crèche may be a necessity. It must be admitted that in many parts of London it is difficult to find reliable women really capable of properly attending to a baby during the day; the crèche, therefore, fills the need. The story of the man and his wife who left the baby at a crèche while they went off to a race meeting may be a picturesque exaggeration, but it is clear from the evidence obtained that such action would be quite possible in many crèches. The crèche system of the present day takes but little into account the desirability of not encouraging women to go out to work.

A special meeting of the Charity Organisation Society was held to consider the facts arising out of the reports of the district committees. Invitations were issued to representatives of all the crèches in London, to medical officers of health, to health societies, and to the National Society of Day Nurseries. There was a large attendance. Dr. Dudfield (Medical Officer of Health for Paddington) and Dr. Sandilands (Medical Officer of Health for Kensington) moved and seconded resolutions which were eventually carried unanimously in the following form:—

(1) All crèches should be registered, and subject to inspection.

(2) Proper arrangements for medical supervision are essential.

(3) Only properly trained persons should be employed as matrons.

(4) Adequate measures should be taken to limit admission to cases of children of women who are compelled to work for their living.

The Charity Organisation Society has been endeavouring for some time past to take some action on the lines of these resolutions. Inquiries have shown that probably the simplest way to secure proper public supervision of crèches and day nurseries would be the insertion of a clause in the Metropolitan Public Health Amendment Act of 1891. Last year, however, did not afford many opportunities for private Bills, and it cannot be said that the prospects this year are very bright. The Local Government Board, however, has been approached, while the Parliamentary representative of the National Society of Day Nurseries has had the matter in hand. It is hoped that there may be an opportunity later for combined action. One of the Society's district committees, in referring to a crèche which is so badly managed as to prove a real danger, asks, "Are there no means of getting this place closed?" Inquiries seem to show that, at present, there are none: future action may make it possible.

